# Heterogeneous timescales are spatially represented

**DOI:** 10.3389/fpsyg.2014.00542

**Published:** 2014-06-03

**Authors:** Mario Bonato, Carlo Umiltà

**Affiliations:** ^1^Department of Experimental Psychology, Ghent UniversityGhent, Belgium; ^2^Department of General Psychology, University of PadovaPadua, Italy

**Keywords:** time, spatial cognition, SNARC effect, mental representations, mental time travel, time perception, mental time line

There is little doubt that cognitive processes involved in perceiving and comparing the duration of the sounds played by a musical instrument are very different from those involved, for instance, in recalling when our latest holidays took place. However, despite their clear difference, we ascribe both processes to the time domain, either in the form of a duration or of a mental time travel. Here we maintain that, despite the several meanings of “time” and its intrinsically heterogeneous nature, a common and somehow surprising characteristic of its representation is its spatial nature. Evidence will be provided suggesting that sensory time (the sounds of the instrument), time travel (last holidays) as well as conceptual time are all spatially represented. Whether this spatial format is the same for all “categories” of time is still unclear, as well as it is unclear whether category-specific spatial layouts exist. The possibility that several heterogeneous aspects of time might be all processed in spatial terms allows to better understand a number of apparently disconnected findings within the vast time domain. We will describe the prominent role played by writing habits in giving a direction to this representation. The possibility will be discussed that a more general tendency exists to represent in a spatial format all ordered sequences, as well as abstract concepts.

The interest on the way humans process and perceive time can be readily explained by the ubiquity of time in human life and by its multifaceted and sometimes elusive characteristics. Moreover, time processing is relevant for several domains, including not only philosophy, linguistics, cognitive science, and neuroscience, but also anthropology, history, and biology. Humans are equipped with a device (or several devices, this does not seem to be currently known) allowing them to deal effectively with all aspects of time, including duration estimates, time travel, and time conceptualization. A key empirical finding supporting our purposes here is that, across a number of formats and paradigms, time processing interacts with spatial processing and with the mechanisms subtending the deployment of spatial attention. This allows to consider the way humans perceive different time durations, and represent heterogeneous time concepts, as spatial in nature. A thorough review on these aspects can be found in Bonato et al. (2012a), where a number of analogies with the spatial representation within the numerical domain -which is also characterized by ordinality- are discussed. The more consistent line of evidence supporting the possibility that time is spatially represented comes from those studies showing an interaction between a time-related characteristic and the response side. Regardless of the aspect of time that is under investigation (perceptual or representational), the typical experimental manipulation requires the use of two stimulus-response mappings, whereby the same time-related stimulus receives a left response in one mapping and a right response in the other. By using two temporal durations Vallesi et al. (2008, exp 1) have shown consistent associations between left side and shorter durations (1 s) and between right side and long durations (3 s). The interaction does not depend on the responding hand but on the side of space where response is performed (exp 3 and 4) and was still present when more durations (six, from 0.5 to 3.5 s) had to be compared (exp 5; see Conson et al., 2008 for similar findings with auditory stimuli). An interaction with lateralized response keys is also found when the to-be-judged temporal references are related to mental time travel/time concepts, (e.g., before-after). These studies typically adopt much longer temporal durations. It is the case, for instance, of the months of the year, whereby faster responses are found when one of the first months (e.g., February) has to be responded to with a left-sided response and one of the last months (e.g., January) with a right-sided response (Gevers et al., 2003). Evidence for the automaticity of this association stems from the fact that it was found also when the task did not involve explicit access to the time aspect (Gevers et al., 2003). Left-right associations can be elicited also when the timeframe in a temporal comparison task extends to several years, as shown by Weger and Pratt (2008) who asked for a comparison of actors who were famous several decades ago (e.g., Charlie Chaplin) vs. those who were famous at the time of testing (e.g., Brad Pitt). Ishihara et al. (2008) provided evidence that left-before vs. right-after associations are present also for brief, sensory stimuli (sounds with a 20 ms duration). In their study, the time of appearance of a target sound after a sequence of seven equally spaced (500 ms interval) sounds had to be compared with the temporal lag of half a second dividing the preceding sounds. Participants showed a preference for right-sided responses when the target sound was presented later in time with respect to the expected interval (delay of 215 ms) and a preference for left-sided responses for a target sound presented 215 ms before the expected timing. The effect disappeared when vertically arranged response keys were adopted. Crucially, a clear left/before right/after association is also found when arbitrary, non-temporal sequences are used (Previtali et al., 2010). In this latter case the temporal aspect is somehow implicit and related to the order of stimuli presentation (time range: several seconds). The association of left responses with past and of right responses with future still persists when past- related vs. future-related tenses and words are presented (Santiago et al., 2007).

An interaction with response side can be also found when merging together (very) different temporal lengths, ranging from a few seconds to several decades (Fuhrman and Boroditsky, 2010). In this study participants had to determine, by means of two lateralized response keys, whether the second of two sequentially presented pictures referred to an event occurring earlier/later in time than the first picture presented. Images represented either brief sequences (seconds-minutes: a banana turning into an empty peel) or long sequences (decades: a boy turning into an adult). English speakers showed a clear left/before right/after preference in 36 out of 38 sequences. By directly comparing the performance of English-speaking people to Hebrew speakers, Fuhrman and Boroditsky (2010) showed that the effects of preferential spatial arrangements with lateralized keys is related to writing directions (see also the seminal study by Tversky et al., 1991). A similar effect of writing habits can be found when temporal durations (1 vs. 3 s) have to be compared (Vallesi et al., in press), once again confirming the commonalities in representing durations and time order. In other words, time representation(s) can be dramatically influenced by cultural factors (Núñez and Cooperrider, 2013). Populations that are not very familiar with reading and writing show a number of different spatial layouts for time (Boroditsky and Gaby, 2010). However, also in Western cultures changes in the postural sway according to a past/backwards and future/forward association has been described (Miles et al., 2010).

In summary, a spatial component (lateralized responses) has been described to be robustly associated with strikingly different time intervals, either with more perceptual or with more conceptual tasks. This suggests that very different time aspects can be all spatially represented. One can make the analogy even stronger by pointing out that this supra-modality of time processing resembles the similarities found for perceived vs. represented spatial locations.

It can be claimed that the ubiquity of these effects can be task-induced rather than reflect a truly spontaneous spatial representation. A line of evidence allowing one to confirm the genuinely spatial origin of time representation can be found in those cases where spatial attention is distorted as a consequence of brain damage. Damage affecting right-hemisphere areas related to spatial processing has been described to induce a spatial bias in a detection task where the cue was a time-related, centrally presented, word (Pun et al., 2010). Moreover, two recent studies with right hemisphere damaged patients have described temporal distortions in the presence of neglect, a neuropsychological syndrome hampering the processing and the internal representation of contralesional space. These findings provide further support for the spatial nature of time representation for both mental time travel (Saj et al., 2014) and temporal durations (Oliveri et al., 2013).

The idea behind Saj et al. (2014) is similar to the rationale of the study by Zorzi et al. (2002), who suggested that the overestimation found in patients with left neglect in a numerical bisection task resembled the length effect neglect patients show in line bisection tasks. In the Saj et al. (2014) study right brain damaged patients with and without spatial neglect encoded behavioral habits an imaginary person used to have in the past (10 years before) or will have in the future (in 10 years). Patients with left hemispatial neglect showed a specific deficit when remembering and attributing items to the past, whereas this “distortion” was absent in right brain damaged patients without neglect and in healthy controls. By using prismatic adaptation Oliveri et al. (2013) crucially demonstrated that the disturbances in the time domain shown by neglect patients result from their impaired spatial processing rather than by other, more general, cognitive impairments (Bonato et al., 2012b). In right brain damaged patients with neglect a leftward attentional deviation reduced the -overall more severe than in patients without neglect- time underestimation deficit. An experimental manipulation (e.g., prismatic adaptation) affecting spatial processing thus influenced also a temporal bisection task. Effects of spatial attention distortions upon time aspects can be found in healthy participants as well (see for instance Vicario et al., 2007; Di Bono et al., 2012).

Which are the cognitive mechanisms subtending time-space interactions and their distortions ? Within the numerical domain Priftis et al. (2006) maintained that the distortions due to neglect in numerical processing reflect impaired access rather than a distorted representation itself. Supporting evidence can be found in Zorzi et al. (2012), where spatio-numerical deficits shown by neglect patients varied according to the task at hand (present in magnitude comparison but absent in parity judgement). Leaving terminological issues aside, the possibility of a deficit in accessing an intact representation does not seem at odds with those studies that have highlighted that working memory for sequences is characterized by a spatial layout. It might then be reasonable to assume that most time-space associations are based on flexible, short-term associations made on the spot according to the task at hand. In turn, this view would be compatible with the findings of van Dijck and Fias (2011), who have described a left to right spatial mapping for ordered items in working memory. This mapping influences not only lateralized responses but also target detection tasks (van Dijck et al., 2013).

Finally, if we reason that perceptual time is in a way represented and conceptualized before response, it seems possible that an even broader tendency to spatially represent all abstract concepts exists. For instance, Chasteen et al. (2010) have shown, through a target detection task, that the concepts of God and Devil produce shifts of attention and lead to faster responses when visual targets are presented at compatible locations with the concepts of God (up/right locations) or Devil (down/left locations). They framed their findings as supporting the link between internal spatial representations and locations where attention is allocated in the external environment. The tendency to represent rather abstract concepts in a spatial format might further extend to a number of representations of the self, in addition to spatial and temporal frames of reference (Parkinson et al., 2014).

## Conflict of interest statement

The authors declare that the research was conducted in the absence of any commercial or financial relationships that could be construed as a potential conflict of interest.
